# Effects of dietary L-glutamine and glutamic acid combination, and whey powder on the performance and nutrient digestion in weaned piglets fed grain-based diets

**DOI:** 10.5713/ab.20.0613

**Published:** 2021-04-23

**Authors:** Jonathan Mádson dos Santos Almeida, Leonardo Augusto Fonseca Pascoal, Jorge Luiz Santos de Almeida, Ricardo Romão Guerra, José Humberto Vilar da Silva, David Rwbystanne Pereira da Silva, Manoel Rosa Silva Neto, Terezinha Domiciano Dantas Martins

**Affiliations:** 1 Post-graduation Program in Animal Science, Federal University of Paraiba, Center of Agrarian Sciences, Areia, 59397000, Brazil; 2 Department of Animal Science, Federal University of Paraíba, Center for Human, Social and Agrarian Sciences, Bananeiras, 58220000, Brazil; 3 Department of Veterinary Sciences, Federal University of Paraíba, Center of Agrarian Sciences, Areia, 59397000, Brazil; 4 Post-graduation Program in Aquaculture, Paulista State University, Unesp Aquaculture Center, São Paulo, 14884900, Brazil

**Keywords:** Functional Amino Acids, Intestine, Swine, Weaning, Whey Powder

## Abstract

**Objective:**

The present study aimed to evaluate the influence of including L-glutamine along with glutamic acid as a supplement in weaned piglets’ diets with and without whey powder.

**Methods:**

Two assays were carried out. A total of 40 piglets ([Landrace×Large White]× Pietrain) weaned at 24 days of age with an initial body weight of 6.6±0.6 kg were used in the first assay, and the following parameters were evaluated: growth performance, the incidence of diarrhea, morphometry, intestinal integrity, and hepatic glycogen index. The animals were then blocked into four groups according to different diets: diet all-grain feeding (G); diet all-grain feeding with whey powder (GW); and with vs without 1% supplementation of the commercial product containing L-glutamine and glutamic acid (A or NA). Whey powder was added according to the stage of life, corresponding to 17%, 10%, and 5%, respectively, in order to meet the need for lactose. The animals were evaluated at 24 to 42 days and at 24 to 55 days of age. The nutrient digestibility for the second assay was carried out by using 24 animals with an average weight of 11.49±1.6 kg, and the same diets were tested.

**Results:**

The supplementation of L-glutamine + glutamic acid or the addition of whey powder in diets for weaned piglets provided (p<0.05) greater feed intake, greater weight gain and improved feed conversion in the initial period (24 to 42 days age). However, in the whole period (24 to 55 days age) only amino acid supplementation affected (p<0.05) growth performance. There was a positive interaction (p<0.05) between the type of diet and L-glutamine + glutamic acid supplementation on villus height, crypt depth and the villus:crypt ratio in the duodenum. In addition, L-glutamine + glutamic acid supplementation reduced (p<0.05) the crypt depth and improved the villus:crypt ratio in the jejunum. The inclusion of whey powder affected (p<0.05) positively the digestibility coefficients analyzed except mineral matter digestibility coeficients. The supplementation of 1% the commercial product composed of L-glutamine and glutamic acid improved (p<0.05) only the digestibility coefficient of crude protein.

**Conclusion:**

These results indicate that supplementation of 1% commercial product containing L-glutamine + glutamic acid in diets for piglets from 24 to 55 days of age, dispenses with the use of whey powder when evaluating growth performance. Amino acid supplementation alone or associated with whey powder affects (p<0.05) positively the indicators of the intestinal integrity.

## INTRODUCTION

The stress caused after early weaning in piglets favors a decrease in voluntary feed intake, especially in the first week after weaning, and may additionally affect the morphometry, intestinal function and immune system by diminishing the digestive capacity with a consequent deficit in absorptive potential [1]. One important tool to minimize the non-desirable effects of weaning is the quality of pre-initial and initial diets. In turn, dairy products stand out for their nutritional qualities and excellent acceptability, and can contribute to improving feed digestibility and maintaining mucosal integrity throughout the small intestine [2]. Furthermore, it has been suggested that the indiscriminate use of antibiotics in animal feed will lead to resistance against pathogenic bacteria which affect animals and humans [3]. As a consequence, several countries have prohibited or will prohibit the use of antibiotics as growth promoters in animal production. Thus, nutritional strategies such as the addition of functional amino-acids to diets such as arginine, glycine, proline, tryptophan, glutamic acid and glutamine have been used to diminish intestinal degeneration after weaning and to promote adequate growth performance [2].

The addition of glutamine to animal diets can improve small intestine morphology, especially in stress situations like weaning. Among other functions, glutamine acts as metabolic fuel for fast renewing cells, and glutamic acid substitutes glutamine in many of its roles, including those for energy generation and amino-acid synthesis [4]. Piglets fed with diets containing 1% glutamine presented higher daily weight gains, feed conversion and villus height (VH) in the jejunum [5].

Although most studies have shown that glutamine and glutamate provide benefits to growth performance index and to intestinal structure, there are few studies on the influence of these amino acids on diets all-grain feeding.

In view of the above, the present study had the objective to evaluate including L-glutamine along with L-glutamic acid in weaned piglets’ diets, with or without whey powder, by measuring the effects on growth performance, incidence of diarrhea, morphometry and intestinal integrity, hepatic glycogen index and nutrient digestibility.

## MATERIALS AND METHODS

The experimental assays were carried out in the Laboratory of Swine Farming of the Department of animal science at the Center for Humanities, Social and Agrarian Sciences in Federal University of Paraíba (Bananeiras, Brazil). Experimental procedures were approved by CEUA/UFPB, under experimental protocol number 044/2015.

### Assay I: Growth performance, morphometry and intestinal integrity

#### Animals and experimental diets

A total of 40 weaned piglets ([Landrace×Large White]×Pietrain) were used. All animals were weaned at 24 days of age with an initial average weight of 6.6±0.6 kg. The piglets were housed in suspended pens with plastic slatted flooring, equipped with nipple waterers.

The animals were distributed in a randomized block design with four treatments and five repetitions, in which the experimental unit was formed by two animals, one male and one female.

The treatments were arranged as follows: diet all-grain feeding (G); diet all-grain feeding with whey powder (GW); and with vs without amino acid supplementation of L-glutamine and acid glutamic (A or NA), totaling four experimental diets. Whey powder was added to meet lactose needs at each stage of life.

The diets were formulated according to Rostagno et al [6] in the following periods: I, from 5 to 9 kg; II, from 9 to 15 kg; and III, from 15 to 30 kg (Table 1). The animals consumed water and feed *ad libitum*. The glutamine and glutamic acid source was the commercial product AminoGut (Ajinomoto, São Paulo, Brazil) which is composed of a minimum of 95% glutamine + glutamic acid containing a mixture of L-glutamine (min 10%) and L-glutamic acid (min 10%).

#### Productive performance and diarrhea incidence

The animals and the leftover feed were weighed at the beginning and at the end of each experimental period, from which the average daily feed intake, average daily gain, and feed:gain of weaned piglets. The growth performance results were analyzed in the following periods: I, from 24 to 42 days of age; and III, from 24 to 55 days of age. The influence of the diets on diarrhea incidence was evaluated in the first 19 days of experiment. To do so, the fecal score was recorded daily in the morning and in the late afternoon. Feces consistency was scored according to visual criteria, always by the same observer. A score of 3 was associated with more liquid feces, considered to be diarrheal.

#### Animal slaughter and histological slide preparation

The animals were slaughtered at the end of the experimental period, animals were fasted for 12 h and weighed before slaughter. Slaughter of the piglets was by electrical stunning followed by exsanguination according to the procedures approved by CEUA/UFPB. The segments of the first portion of duodenum, middle portion of jejunum and liver were subsequently collected after slaughter and immersed into formalin solution immediately for later histological evaluations. The samples were dehydrated in increasing solutions of alcohol, diaphanized in xylene and paraffin-embedded according to the protocol described by Yoon et al [7]. Next, 5 μm-sections were cut in microtome and stained for histological analysis.

#### Morphometry and intestinal integrity

The slides were stained with hematoxylin and eosin to evaluate intestinal morphometry. The VH and crypt depth (CD) were measured. Next, the villus/crypt ratio (VH/CD), mucosal thickness, and villus width (VW) were calculated from these data. The goblet cell index was then measured by using Periodic Acid Schiff (PAS) stain, an area of 10,000 μm was measured and then the amount of globet cells present in the intestinal villus was contend. Absorptive area was determined from the VH and VW.

The nuclear protein proliferating cell nuclear antigen (PCNA-mitosis rate) and the cell death by apoptosis (caspase) were evaluated in duodenum and jejunum fragments. The protocol used for all antibodies was based on the immunohistochemistry technique [8].

The apoptosis rate in the villus cells of the first portion of duodenum and middle portion of jejunum was evaluated by the anti-neutrophil cytoplasmic antibody positivity. Thus, positivity scores were attributed for the 20 photomicrographs of each treatment according to the following scale: 0 (absence of positivity), 1 (low positivity), 2 (mild positivity), and 3 (intense positivity), as reported in the semi-quantitative score methodology of Ishak et al [9], with modifications. The crypts were analyzed and randomly measured to evaluate the cell mitosis rate in first portion of duodenum and middle portion of jejunum, making a total of 10,000 μm of epithelium per treatment. The epithelial cells were quantified according to the number of anti-PCNA+ nuclei.

The readings and digitalization of histological slides were carried out by using an Olympus BX53 light microscope with the lens objective at 40x, and a Zeiss Axio camera coupled to a computer equipped with the CellSens Dimension software program for image acquisition.

#### Evaluation of hepatic glycogen

The slides containing the hepatic tissue were stained with PAS to evaluate the glycogen index. The photomicrographs were then analyzed by using a score for the glycogen deposit (glycogen score) degree as follows: 0 (absence of positivity), 1 (low positivity), 2 (mild positivity), and 3 (intense positivity), according to the semi-quantitative score methodology proposed by Ishak et al [9], with modifications.

The readings and digitalization of histological slides were performed by using an Olympus BX53 light microscope with the lens objective at 40x, and a Zeiss Axio camera coupled to a computer equipped with the CellSens Dimension software program for image acquisition.

### Assay II: Digestibility of experimental diets

#### Location, animals and experimental diets

A total of 24 castrated male piglets ([Landrace×Large White]×Pietrain) with an average initial weight of 11.49±1.6 kg and 42 days of the age were used. The animals were distributed in a randomized block design with four treatments and six replications, in which the experimental unit was formed by a male animal. These were transferred to a masonry shed and housed in cages for metabolic studies. A period of twelve days was established, from which the first seven days served for adaptation to the cages and rations, and to determine consumption, whereas feces and urine were then collected in the last five days. The animals received the same experimental diets of assay I, filling the nutritional requirement for the period (Table 1).

#### Digestibility

First, 1% iron oxide was used as fecal marker to set the beginning and end of collection. The daily urine was collected in a plastic bucket with a filter containing 20 mL of hydrochloric acid (HCl) at a ratio of 1:1. The produced volume was measured and an aliquot of 20% was stored in a freezer. The feces and urine of each animal were thawed, homogenized and analyzed at the end of experimental period.

The ration, feces and urine samples were analyzed according to the procedures described by AOAC [10] for determining dry matter, crude protein, mineral matter and organic matter. The crude energy was determined using an adiabatic bomb calorimeter (model 6100, Parr Instruments Co., San Franscisco, CA, USA) from which the metabolizable energy was obtained. The nutrient digestibility was determined as digestibility coefficients and energy metabolizability coefficient. These values were calculated according to Aldeola et al [11].

### Statistical analysis

The data were evaluated using a 2×2 factorial scheme (two diets: diet all-grain feeding or diet all-grain feeding with whey powder) and supplemented or not with L-glutamine + glutamic acid. The error normality was observed by the Cramer-von Misses test. Data were subjected to analysis of variance using the general linear models procedure in the SAS statistical program (SAS University, 2018). The factors were subsequently compared by the Tukey test when the interaction was significant, and the Student’s t-test for the isolated factors. Non-parametric statistics were used to evaluate the diarrhea incidence, cell apoptosis and hepatic glycogen, and the observations were compared using the Wilcoxon test (5%) among the isolated factors (diet and supplementation).

## RESULTS

### Assay I

The L-glutamine with glutamic acid supplementation in diets containing or not containing whey powder affected (p<0.05) the productive performance variables of weaned piglets (Table 2).

The type of diet and the L-glutamine and glutamic acid supplementation affected (p<0.05) the analyzed variables from 24 to 42 days of age. Animals that received the diet all-grain feeding showed lower daily feed intake, lower daily weight gain and lower feed conversion when compared to those that consumed the diet containing whey powder. It was observed that supplementation with the product containing glutamine associated with glutamic acid improved (p<0.05) the growth performance parameters in weaned piglets.

An effect (p<0.05) of diet and L-glutamine and glutamic acid supplementation on daily feed intake was observed for data related to the whole period from 24 to 55 days of age, thus indicating lower intake when animals received diet all-grain feeding diets and without supplementation of the commercial product. Furthermore, the animals that consumed the diets without amino acid supplementation showed (p<0.05) the lowest weight gains and worsening in feed conversion. There was no effect (p>0.05) of experimental diets on the diarrhea incidence.

The L-glutamine and glutamic acid supplementation in diets containing (or not) whey powder affected (p<0.05) the variables related to intestinal integrity and cell mitosis in the duodenum of weaned piglets (Table 3). There was an interaction effect between the type of diet and the supplementation containing the amino acids in the VH, CD, VH/CD ratio, and VW variables. Animals that consumed the diet all-grain feeding without glutamine and glutamic acid had lower VH and VH/CD, greater CD and VW. The mucosa thickness, absorptive area, goblet cell count and cell mitosis rate variables were influenced (p<0.05) by the type of diet, for which the animals that consumed the diet all-grain feeding showed higher values.

The L-glutamine and glutamic acid supplementation affected (p<0.05) the CD and the VH/CD in the weaned piglets’ jejunum, with greater depth and a lower ratio being verified in the animals that consumed the diet without supplementation (Table 4). There was an interaction effect between the type of diet and the supplementation (or not) of L-glutamine and glutamic acid on the goblet cell count and the cell mitosis rate, in which the animals that consumed the diet containing whey powder and L-glutamine associated with glutamic acid had the lowest values.

The apoptosis rate in the duodenum was influenced (p< 0.05) by the type of diet, in which animals that consumed the diet all-grain feeding had a higher apoptosis rate when compared to animals that consumed the diet with whey powder (Table 3).

The L-glutamine and glutamic acid supplementation affected (p<0.05) the deposition of hepatic glycogen in weaned piglets, in which animals that consumed the diets with amino acid supplementation had a higher glycogen index (Table 5).

### Assay II

The type of diet influenced (p<0.05) the digestibility coefficients of dry matter, organic matter, crude protein, crude energy, and the energy metabolizability coefficient of weaned piglets, with the lowest digestibility results for animals who consumed the diet all-grain feeding (Table 6). The digestibility coefficient of crude protein was also influenced by the L-glutamine and glutamic acid supplementation, with an improvement in the coefficient for animals that received supplementation.

## DISCUSSION

Animals that consumed the diet all-grain feeding (G) mainly composed of corn and soybean meal presented worsening in the performance variables in initial and whole periods when compared with the animals that received the diets with the L-glutamine and glutamic acid supplementation containing (or not) whey powder. This can be explained by the fact that the piglets’ digestive system is adapted from birth to weaning to secrete the digestive enzymes which digest lactose in milk in greater quantity, but not other ingredients, especially those of plant origin [12].

In addition, ingredients of grain origin (such as soy naturally) present compounds which act as natural plant protection known as anti-nutritional factors, such as trypsin and chymotrypsin inhibitors, which inhibit protein digestion; lectins, whose main action mode combine with intestinal wall cells and thereby cause non-specific interference in the absorption of nutrients; allergic factors (glycinin and β-conglycinin), which reduce nutrient absorption and cause deleterious effects on the microvilli of the small intestine; and there are also soluble non-starch polysaccharides, which cause a decrease in animal performance, and which can remain present even after thermal processing [13].

In turn, the inclusion of the product containing L-glutamine associated with glutamic acid in diets containing (or not) whey powder reduced the deleterious effect of the diet all-grain feeding. This was probably due to the fact that glutamine has important functions, especially in the gastrointestinal tract, being used as an energy fuel by rapidly dividing cells such as intestinal mucosa and immune system cells [14].

Likewise, glutamic acid from the diet is an important fuel for epithelial cells, in addition to being involved in the excitatory neurotransmission of efferent vagal stimulation and in nutrient detection [15].

In this context, results found by Cabrera et al [16] showed that supplementation with 1% L-Glutamine or 0.88% aminoGut (commercial product with at least 10% glutamine and 10% glutamic acid) improved the feed conversion rate in the first three weeks after weaning; however, no difference was found for other performance variables. When supplemented with glutamine and glutamic acid at levels of 0.1% glutamine + 0.9% glutamic acid; 0.2% glutamine + 0.8% glutamic acid; 1% glutamine and 1% glutamic acid in the diet of piglets with an average weight of 9.22 kg, He et al [5] observed that piglets weaned at 28 days consumed the diet with 1% glutamine more and showed better weight gain in the 28 days of the experiment when compared with the animals of the control diet.

In an experiment lasting 14 days which analyzed the performance of piglets weaned at 18 days and exposed to a 12-hour transport simulation, 0.20% glutamine supplementation provided an increase in feed intake after the 10th day and greater body weight after the 13th day when compared to treatments with or without antibiotics [17].

In contrast to these results, Rodrigues et al [18] did not observe an improvement in the performance of piglets recently weaned and fed diets containing 0.8% L-glutamine associated with L-glutamic acid with or without the addition of valine in the period from 24 to 46 days of age.

Regarding diarrhea incidence, there was no effect of the treatments tested in this study. Weaning causes a harmful effect on the intestinal barrier function, often observed by breaking it, providing increased permeability to toxins, bacteria and antigen-associated foods, which penetrate the intestinal epithelium and result in inflammation, malabsorption, diarrhea and reduced growth [19]. Post-weaning diarrheal syndrome is related to the exacerbated multiplication of pathogenic bacterial strains in the piglet intestine and is associated with nutritional, management and environmental factors [20].

In addition, diets for piglets in the post-weaning period have a high crude protein level, which can undergo microbial fermentation when not fully digested, favoring the occurrence of diarrhea due to increased proliferation of pathogenic bacteria [20]. Therefore, the fact that all animals consumed isoproteic diets and were subjected to similar handling and facilities may have influenced the results of the present study. These results corroborate those found by Rodrigues et al [18] who did not observe the influence of diets supplemented with L-glutamine associated with L-glutamic acid with or without the addition of valine on diarrhea incidence in piglets from 24 to 46 days of age.

However, supplementation with 1% L-glutamine and glutamic acid in the diet of piglets weaned at 21 days of age caused lower diarrhea incidence when compared to animals which consumed the control diet [21].

The diet all-grain feeding mainly composed of corn and soybean meal without the addition of the product containing glutamine and glutamic acid negatively affected the parameters related to intestinal integrity and cell proliferation rate (cell mitosis). One of the probable causes of this to explain this result is that the piglet digestive system undergoes modifications in weaning until it is prepared for digesting ingredients of plant origin [12]. Thus, it is essential that the gastrointestinal tract identify the changes that the new diet causes so that it is able to promote changes in pH, enzyme secretion and motility, with consequent improvement of digestive and absorptive processes [22].

Another factor which can compromise intestinal integrity is the feed consumption. Low feed consumption can cause inhibitory morphometric changes, since the intestinal segments need to obtain nutrients in sufficient quantity to meet the demands for mucosal protein synthesis and growth. These modifications include shortening and modification of the villus structure, hyperplasia of the crypt cells, in addition to functional changes in the intestine [23] decreased VH/CD, increased goblet cell count, which is closely related to epithelium protection, and an increase in the mitosis rate in the intestinal epithelium through cell turnover due to a higher desquamation rate.

However, it was found that supplementation with the product containing L-glutamine and glutamic acid on diet all-grain feeding promoted an increase in VH, a decrease in CD and an improvement in the VH/CD, which provides greater contact area with nutrients and consequently greater absorption of them. Glutamine is the main source of energy for intestinal tissue, with 67% of dietary glutamine being used by mucous cells, intestinal cells and bacteria. This amino acid provides essential energy for cell multiplication and favors biosynthesis of nucleic acids such as purines and pyrimidines, which play a fundamental role in maintaining intestinal integrity [14; 24]. Likewise, glutamic acid has been shown to exert numerous functions in the metabolism of nutrients. Its importance is due to the fact that it is the main oxidative fuel for intestinal cells, and a precursor for the synthesis of important cellular antioxidants such as glutathione, arginine and proline which compose glycoproteins present in intestinal mucus [24].

In this context, in a study carried out with 1% glutamine supplementation in the diet of piglets weaned at 21 days of age and slaughtered at 28 days of age, the VH and the VH/CD (p<0.05) of the pigs increased in the jejunum when compared with those that were not supplemented [25]. In the same sense, in evaluating the supplementation of 0.20% glutamine, there was an increase (p<0.05) in the VH in the duodenum and the VH/CD in the duodenum and jejunum of piglets when compared to the treatment without the use of antibiotics [17].

On the other hand, in evaluating glutamine supplementation associated with L-glutamic acid at levels of 0.1% glutamine + 0.9% glutamic acid; 0.2% glutamine + 0.8% glutamic acid; 1% glutamine and 1% glutamic acid, He et al [5] found that they did not affect (p>0.05) VH, CD, or VH/CD of duodenum, jejunum or ileum in piglets slaughtered at 28 days. In another study testing different diets (control; with 1% glutamic acid; 1% glutamine and 1% nucleotides) for piglets, Amorim et al [26] did not observe an effect (p>0.01) on VH or VH/CD; however, it was found that there was a decrease (p<0.01) in the CD in the group supplemented with 1% glutamine when compared to the group that received 1% nucleotide.

There was a higher apoptosis rate in the duodenum in the animals that received the totally diet all-grain feeding when compared to those which consumed the diet containing the whey powder, which may have been responsible for the changes in the morphometric parameters. However, there was a reduction in the apoptosis rate due to the inclusion of dairy products in the diet. Lactose present in dairy products acts by maintaining the integrity of the intestinal mucosa [2] and in turn may have an inhibitory role on the cell apoptosis rate.

The higher deposition of hepatic glycogen in animals that consumed diets supplemented with L-glutamine and glutamic acid may be related to the participation of this amino acid in energy metabolism, as evidenced by the participation in the synthesis of amino sugars, purines and pyrimidines which are part of A, ATP, and NADH coenzyme molecules which are direct participants in the Krebs Cycle. Linked to this, glycogen synthesis is stimulated by glutamine, which has a direct role in glycogenesis due to its ability to increase glycogen synthetase enzyme activity, which favors forming glycogen in the liver [29]. In addition, glutamic acid is used by succinic, fumaric, malic, oxaloacetic and other acids in its metabolism for the synthesis of glycogen which can be stored (in most cases) in the liver tissue [30].

The consumption of a totally diet all-grain feeding constrained the digestibility coefficients of nutrients, probably due to the low activity of specific enzymes for degrading ingredients of plant origin. The complete development of the enzymatic system usually takes place until the eighth week of life in piglets, so the gastrointestinal tract produces insufficient carbohydrates, proteases and other enzymes which act in digesting ingredients which are usually used in starter diets in the post-weaning period, especially those of plant origin [31].

In this sense, lactose present in dairy products is widely used in composing diets for piglets right after the weaning period, as it acts in improving the feed acceptability [32] and increases consumption of dry matter, nitrogen digestibility and promptly provides energy to the tissues [33] in addition to favoring animal feed consumption.

The improvement in the digestibility coefficients of the diets containing whey powder (GW) varied from 1.02% to 1.76%, which may explain the non-improvement in the weight gain and feed conversion of the animals in the period from 24 to 55 days, receiving GW diets compared to grain-based (G) diets, another aspect that may be related to the method used to determine the digestibility of diets, in which the animal receives a restricted amount of feed, based on its metabolic weight, which it enhances the use of nutrients [34] and there is a reduction in energy losses [35] differently from animals that receive feed at will, which increases their maintenance needs due to increased consumption and is necessarily associated with compensatory gain [35].

The highest digestibility coefficient of crude protein observed was when supplementation of the product containing L-glutamine and glutamic acid occurred; this can be explained by the great importance that glutamine has in metabolic processes. It is an indispensable amino acid for the growth of most cells and tissues, and glutamine can act as a metabolic regulator to increase synthesis and reduce protein catabolism under conditions of high protein degradation which include periods of stress (such as weaning), as well as periods of rapid tissue growth and diseases [36].

These results indicate that supplementation of 1% commercial product containing L-glutamine + glutamic acid in diets for piglets from 24 to 55 days of age, dispenses with the use of whey powder when evaluating growth performance. Amino acid supplementation alone or associated with whey powder affects (p<0.05) positively the indicators of the intestinal integrity.

## Figures and Tables

**Table 1:** Ingredients and chemical composition of the diets for piglets^1)^

Items	24 to 35 of age (5 to 9 kg)		36 to 42 of age (9 to 15 kg)		43 to 65 of age (15 to 30 kg)	
		
	G^2)^	GW^3)^	G^2)^	GW^3)^	G^2)^	GW^3)^
Ingredients						
Yellow corn	57.60	45.00	57.00	48.62	65.00	60.00
Soybean meal	30.47	28.04	33.76	32.68	29.82	29.47
Whey powder	-	17.65	-	10.29	-	5.00
Soy oil	5.10	4.89	4.70	4.50	1.39	1.60
Sugar	1.53	-	-	-	-	-
Limestone	0.53	0.63	0.63	0.42	0.76	0.79
Dicalcium phosphate	2.14	1.54	1.84	1.91	1.48	1.31
AminoGut^4)^	-	-	-	-	-	-
L-lysine HCl (98.5%)	0.76	0.87	0.50	0.44	0.28	0.24
DL-methionine (99%)	0.28	0.32	0.17	0.17	0.05	0.06
L-tryptophan (10%)	0.06	0.06	0.02	0.01	-	-
L-threonine (98.5%)	0.34	0.06	0.20	0.18	0.07	0.06
Mineral and vitamin supplement^5)^	0.50	0.50	0.50	0.50	0.50	0.50
Salt	0.61	0.18	0.48	0.26	0.40	0.30
BHT	0.02	0.02	0.02	0.02	0.02	0.02
Inert^6)^	0.06	-	0.18	-	0.23	0.65
Total	100.00	100.00	100.00	100.00	100.00	100.00
Calculated values (%)						
Metabolizable energy (MJ/kg)	14.22	14.22	14.12	14.12	13.51	13.51
Crude protein	20.00	20.00	21.00	21.00	19.50	19.50
Calcium	0.85	0.85	0.82	0.82	0.77	0.77
Lactose	-	12.35	-	7.20	-	3.50
Available phosphorous	0.45	0.45	0.45	0.45	0.38	0.38
Digestible tryptophan	0.26	0.26	0.24	0.24	0.24	0.24
Digestible lysine	1.45	1.45	1.33	1.33	1.08	1.08
Digestible methionine	0.55	0.54	0.47	0.47	0.33	0.33
Digestible methionine + cystine	0.82	0.81	0.74	0.74	0.60	0.60
Digestible threonine	0.91	0.91	0.84	0.84	0.68	0.68

1) Ingredients nutritional values were according to the recommendations of Rostagno et al [6].

2) G, diet all-grain feeding;

3) GW, diet all-grain feeding with whey powder; There was supplementation with 1% AminoGut®.

4) AminoGut®, commercial product composed by minimum of 95% glutamine + glutamic acid. in both diets totaling four experimental diets.

5) Mineral supplement: iodine, 140 μg; selenium, 300 μg; manganese, 10 mg; Zinc, 100 mg; copper, 10 mg; Iron, 99 mg. Vitamin supplement: vitamin A, 4.000 IU; vitamin D3, 220 IU; vitamin E, 22 mg; vitamin K, 0.5 mg; vitamin B2, 3.75 mg; vitamin B12, 20 mg; calcium pantothenate, 12 mg; niacin, 20 mg; choline, 400 mg.

6) Sand.

**Table 2:** Influence of supplementation of L-glutamine + L-glutamic acid whey powder in weaned piglets

Item		NA^1)^	A^2)^	Mean	Diet^3)^	Amin^4)^	Diet×Amin^5)^	MSE^6)^
1st period (24 to 42 days of age)								
ADFI (kg)	G^7)^	0.349	0.382	0.366^B^	0.037	0.028	0.656	0.037
	GW^8)^	0.392	0.398	0.395^A^				
	Mean	0.371^b^	0.391^a^					
ADG (kg)	G	0.228	0.247	0.237^B^	0.030	0.049	0.327	0.031
	GW	0.252	0.284	0.270^A^				
	Mean	0.240^b^	0.268^a^					
F:G	G	1.533	1.547	1.540^A^	0.043	0.012	0.147	0.152
	GW	1.579	1.418	1.490^B^				
	Mean	1.556^a^	1.475^b^					
Whole period (24 to 55 days of age)								
ADFI (kg)	G	0.563	0.610	0.586^B^	0.042	0.023	0.637	0.058
	GW	0.602	0.618	0.611^A^				
	Mean	0.582^b^	0.614^a^					
ADG (kg)	G	0.351	0.416	0.384	0.924	0.050	0.428	0.046
	GW	0.373	0.387	0.381				
	Mean	0.362^b^	0.400^a^					
F:G	G	1.604	1.513	1.559	0.778	0.053	0.808	0.149
	GW	1.607	1.601	1.604				
	Mean	1.606^a^	1.562^b^					

ADFI, average daily feed intake; ADG, average daily gain; F:G, feed:gain.

1) NA, diet not supplementation of AminoGut.

2) A, supplemented diet with 1% AminoGut.

3) Value P for diet.

4) Value P for supplementation with 1% AminoGut, commercial product composed by minimum of 95% glutamine + glutamic acid.

5) Value P for interaction diet and AminoGut.

6) MSE, mean standard error.

7) G, diet all-grain feeding.

8) GW, diet all-grain feeding with whey powder.

a,b Means with different letters in the same row differ (p<0.05).

A,B Means with different letters in the same column differ (p<0.05).

**Table 3:** Intestinal morphometry, mitosis rate and apopitosis in the duodenal epithelium of piglets fed diets supplemented with L-glutamine + L-glutamic acid in diets with or without whey powder

Item		Duodenum						

		NA^1)^	A^2)^	Mean	Diet^3)^	Amino^4)^	Diet×Amin^5)^	MSE^6)^
VH (μm)	G^7)^	276.08^Ab^	367.05^Aa^	321.56	0.934	0.012	0.007	34.324
	GW^8)^	322.20^Aa^	318.38^Aa^	320.29				
	Mean	299.14	412.71					
CD (μm)	G	227.86^Aa^	121.32^Ab^	174.59	0.005	0.001	0.001	22.583
	GW	130.58^Ba^	130.32^Aa^	130.45				
	Mean	179.22	125.82					
VH/CD ratio	G	1.26^Ab^	3.03^Aa^	2.74	0.025	0.001	0.001	0.2861
	GW	2.47^Aa^	2.46^Aa^	2.48				
	Mean	1.86	2.74					
VW (μm)	G	216.32^Aa^	149.08^Ab^	182.70	0.005	0.003	0.001	19.732
	GW	150.58^Ba^	154.46^Aa^	152.52				
	Mean	183.45	151.77					
MT (μm)	G	503.92	628.36	566.14^A^	0.026	0.213	0.185	102.421
	GW	452.78	448.68	450.73^B^				
	Mean	478.35	538.52					
AA (μm)	G	59,359.80	54,670.24	57,015.02^A^	0.044	0.608	0.475	8,363.317
	GW	48,446.40	49,231.80	48,839.10^B^				
	Mean	53,903.10	51,951.02					
GC^9)^ (n°)	G	98.00	90.00	94.00^A^	0.002	0.139	0.486	7.777
	GW	82.00	79.00	80.50^B^				
	Mean	90.00	84.50					
Mitosis (n°)	G	883.99	981.32	932.65^A^	0.001	0.615	0.102	95.246
	GW	785.32	731.97	758.64^B^				
	Mean	834.65	856.64					
Apopt^10)^	G	19.85	27.85	23.85^B^	0.01	0.212	-	6.560
	GW	18.00	16.30	17.15^B^				
	Mean	22.08	18.93					

VH, villus height; CD, crypt depth; VH/CD, villus height:crypt depth ratio; VW, villus width; MT, mucosal thickness; AA, absorptive area; GC, goblet cell count.

1) NA, diet not supplementation of AminoGut.

2) A, supplemented diet with 1% AminoGut.

3) Value P for diet.

4) Value P for supplementation with 1% AminoGut, commercial product composed by minimum of 95% glutamine + glutamic acid.

5) Value P for interaction diet and aminoGut.

6) MSE, mean standard error.

7) G, diet all-grain feeding.

8) GW, diet all-grain feeding with whey powder.

9) An area of 10,000 μm was measured and then the amount of globet cells present in the intestinal villus was contend

10) Cell apoptosis (positivity scores).

a,b Means with different letters in the same row differ (p<0.05).

A,B Means with different letters in the same column differ (p<0.05).

**Table 4:** Intestinal morphometry, mitosis rate and apopitosis in the jejunum epithelium of piglets fed diets supplemented with L-glutamine + L-glutamic acid in diets with or without whey powder

Item		Jejunum						

		NA^1)^	A^2)^	Mean	Diet^3)^	Amino^4)^	Diet×Amino^5)^	MSE^6)^
VH (μm)	G^7)^	434.72	453.38	444.05	0.732	0.788	0.317	31.641
	GW^8)^	444.54	433.66	439.10				
	Mean	439.63	443.52					
CD (μm)	G	265.08	219.16	242.12	0.174	0.027	0.958	41.952
	GW	239.00	191.08	215.04				
	Mean	252.04^a^	205.12^b^					
VH/CD ratio	G	1.68	2.12	1.90	0.274	0.020	0.900	0.351
	GW	1.88	2.28	2.08				
	Mean	1.78^b^	2.20^a^					
VW (μm)	G	116.52	120.82	118.67	0.151	0.462	0.867	16.306
	GW	104.12	110.90	107.51				
	Mean	110.32	115.86					
MT (μm)	G	699.82	672.54	686.18	0.231	0.116	0.547	56.894
	GW	683.50	624.74	654.12				
	Mean	691.66	648.64					
AA (μm)	G	50,542.60	54,658.20	52,600.40	0.218	0.435	0.823	8,903.612
	GW	46,274.20	48,578.40	47,426.30				
	Mean	48,408.40	51,618.30					
GC^9)^ (n°)	G	74.00^A^^a^	85.00^A^^a^	79.50	0.003	0.746	0.058	10.155
	GW	61.00^A^^a^	53.00^B^^a^	57.00				
	Mean	67.50	69.00					
Mitosis (n°)	G	745.29^A^^a^	886.65^A^^a^	815.97	0.008	0.346	0.028	91.296
	GW	718.66^A^^a^	657.32^B^^a^	687.99				
	Mean	731.98	771.98					
Apopt^10)^	G	17.85	23.95	20.90	0.807	0.330	-	9.742
	GW	20.10	20.10	20.10				
	Mean	22.03	18.98					

VH, villus height; CD, crypt depth; VH/CD, villus height:crypt depth ratio; VW, villus width; MT, mucosal thickness; AA, absorptive area; GC, goblet cell count.

1) NA, diet not supplementation of AminoGut.

2) A, Supplemented diet with 1% AminoGut.

3) Value P for diet.

4) Value P for supplementation with 1% AminoGut, commercial product composed by minimum of 95% glutamine + glutamic acid.

5) Value P for interaction diet and AminoGut.

6) MSE, mean standard error.

7) G, diet all-grain feeding.

8) GW, diet all-grain feeding with whey powder.

9) an area of 10,000 μm was measured and then the amount of globet cells present in the intestinal villus was contend.

10) Cell apoptosis (positivity scores).

a,b Means with different letters in the same row differ (p<0.05).

A,B Means with different letters in the same column differ (p<0.05).

**Table 5:** Hepatic glycogen storage in piglets fed diets supplemented with L-glutamine + L-glutamic acid, containing or not whey powder

Item		NA^1)^	A^2)^	Mean	Diet^3)^	Amino^4)^	MSE^5)^
Glycogen score	G^6)^	1.35	1.55	1.45	0.281	0.001	0.619
	GW^7)^	1.10	2.10	1.60			
	Mean	1.22^b^	1.82^a^				

1) NA, diet not supplementation of AminoGut.

2) A, supplemented diet with 1% AminoGut.

3) Value P for diet.

4) Value P for supplementation with 1% AminoGut, commercial product composed by minimum of 95% glutamine + glutamic acid;

5) MSE, mean standard error.

6) G, diet all-grain feeding.

7) GW, diet all-grain feeding with whey powder.

a,b Means with different letters in the same row differ (p<0.05).

A,B Means with different letters in the same column differ (p<0.05).

**Table 6:** Digestibility coeficients and energy metabolizability coefficient of piglets supplemented with L-glutamine + L-glutamic acid with or without whey powder

Item		NA^1)^	A^2)^	Mean	Diet^3)^	Amino^4)^	Diet×amino^5)^	MSE^6)^
DMDC	G^7)^	87.53	87.87	87.70^B^	0.0422	0.2118	0.5914	1.119
	GW^8)^	88.29	89.14	88.72^A^				
	Mean	87.91	88.51					
OMDC	G	88.91	89.10	89.00^B^	0.0148	0.1599	0.3345	0.962
	GW	89.60	90.57	90.09^A^				
	Mean	89.25	89.84					
MMDC	G	69.25	70.72	69.98	0.2291	0.9021	0.4013	4.874
	GW	68.47	66.50	67.49				
	Mean	68.86	68.61					
CPDC	G	84.81	86.28	85.55^B^	0.0155	0.0105	0.5291	1.582
	GW	86.16	88.46	87.31^A^				
	Mean	85.49^b^	87.37^a^					
CEDC	G	88.64	88.62	88.63^B^	0.0236	0.3398	0.3213	1.068
	GW	89.29	90.17	89.73^A^				
	Mean	88.96	89.39					
EMC	G	87.94	87.90	87.92^B^	0.0258	0.3892	0.3376	1.082
	GW	88.60	89.43	89.01^A^				
	Mean	88.27	88.66					

DMDC, dry matter digestibility coeficient; OMDC, organic matter digestibility coeficient; MMDC, mineral matter digestibility coeficient; CPDC, crude protein digestibility coefficient; CEDC, crude energy digestibility coefficient; EMC, energy metabolizability coefficient.

1) NA, diet not supplementation of AminoGut.

2) A, supplemented diet with 1% AminoGut.

3) Value P for diet.

4) Value P for supplementation with 1% AminoGut, commercial product composed by 10% of L-glutamine + 10% of L-glutamic acid.

5) Value P for interaction diet and AminoGut.

6) MSE, mean standard error.

7) G, diet all-grain feeding.

8) GW, diet all-grain feeding with whey powder.

a,b Means with different letters in the same row differ (p<0.05).

A,B Means with different letters in the same column differ (p<0.05).

## References

[1] Shan Y, Shan A, Li J, Zhou C (2012). Dietary supplementation of arginine and glutamine enhances the growth and intestinal mucosa development of weaned piglets. Livest Sci.

[2] Pierce KM, Callan JJ, McCarthy P, O’Doherty JV (2005). Performance of weanling pigs offered low or high lactose diets suplplemented with avilamycin or inulin. Anim Sci.

[3] Van der Fels-Klerx HJ, Puister-Jansen LF, Van Asselt ED, Burgers SLGE (2011). Farm factors associated with the use of antibiotics in pig production. J Anim Sci.

[4] Lobley GE, Hoskin SO, McNeil CJ (2001). Glutamine in animal science and production. J Nutr.

[5] He J, Feng GD, Ao X (2016). Effects of L-glutamine on growth performance, antioxidant ability, immunity and expression of genes related to intestinal health in weanling pigs. Livest Sci.

[6] Rostagno HS, Albino LFT, Donzele JL (2011). Brazilian tables for Poultry and swine: food composition and nutritional requirements.

[7] Yoon JH, Ingale SL, Kim JS (2012). Effects of dietary supplementation of antimicrobial peptide-A3 on growth performance, nutrient digestibility, intestinal and fecal microflora and intestinal morphology in weanling pigs. Anim Feed Sci Technol.

[8] Terzian ACB, Zuccari DAPC, Pereira RS (2007). Evaluation of caspase-3 and Ki-67 as a prognostic markers in canine mammary tumors. Braz J Vet Res Anim Sci.

[9] Ishak K, Baptista A, Bianchi L (1995). Histological grading and staging of chronic hepatitis. J Hepatol.

[10] Latimer GW, AOAC International (2012). Official methods of analysis of AOAC International.

[11] Aldeola O, Lewis AJ, Southern LL (2001). Digestion and balance techniques in pigs. Swine nutrition.

[12] Makkink CA, Berntsen PJM, Kamp BML, Kemp B, Verstegen MWA (1994). Gastric protein breakdown and pancreatic enzyme activities in response to two different dietary protein sources in newly weaned pigs. J Anim Sci.

[13] Lima DM, Monteiro PBS, Rangel AHN, Maciel MV, Oliveira SEO, Freire DA (2010). Fatores anti-nutricionais para ruminantes. Acta Vet Bras.

[14] Molino JP, Donzele JL, Oliveira RFM (2012). L-glutamine and L-glutamate in diets with different lactose levels for piglets weaned at 21 days of age. R Bras Zootec.

[15] Janeczko MJ, Stoll B, Chang X, Guan X, Burrin DG (2007). Extensive gut metabolism limits the intestinal absorption of excessive supplemental dietary glutamate loads in infant pigs. J Nutr.

[16] Cabrera RA, Usry JL, Arrellano C (2013). Effects of creep feeding and supplemental glutamine or glutamine plus glutamate (AminoGut) on pre- and post-weaning growth performance and intestinal health of piglets. J Anim Sci Biotechnol.

[17] Johnson JS, Lay DC (2017). Evaluating the behavior, growth performance, immune parameters, and intestinal morphology of weaned piglets after simulated transport and heat stress when antibiotics are eliminated from the diet or replaced with L-glutamine. J Anim Sci.

[18] Rodrigues ND, Bridi AM, Nogueira E (2015). Supplementation of diets for weaned piglets withL-Valine and L-Glutamine+ L-Glutamic acid. B Indústr Anim.

[19] Campbell JM, Crenshaw JD, Polo J (2013). The biological stress of early weaned piglets. J Anim Sci Biotechnol.

[20] Lima GJMM, Mores N, Sanches RL (2009). As diarreias nutricionais na suinocultura. Acta Sci Vet.

[21] Teixeira AO, Nogueira ET, Kutschenko M, Rostagno HS, Lopes DC (2014). Inclusion of glutamine associated with glutamic acid in the diet of piglets weaned at 21 days of age. Rev Bras Saúde Prod Anim.

[22] Lallès JP, Bosi P, Smidt H, Stokes CR (2007). Nutritional management of gut health in pigs around weaning. Proc Nutr Soc.

[23] Lin M, Zhang B, Yu C (2014). L-Glutamate supplementation improves small intestinal architecture and enhances the expressions of jejunal mucosa amino acid receptors and transporters in weaning piglets. Plos One.

[24] Wu G, Bazer FW, Johnson GA (2011). Triennial Growth Symposium: Important roles for L-glutamine in swine nutrition and production. J Anim Sci.

[25] Hao W, Chen Z, Guoyao W (2015). glutamine enhances tight junction protein expression and modulates corticotropin-releasing factor signaling in the jejunum of weanling piglets. J Nutr.

[26] Amorim AB, Berto DA, Saleh MAD, Miassi GM, Ducatti C (2017). Dietary glutamine, glutamic acid and nucleotides increase the carbon turnover (δ ^13^C) on the intestinal mucosa of weaned piglets. Animal.

[27] Mendonça RZ, Arronzio SJ, Antoniazzi MM (2002). Metabolic active-high density VERO cell cultures on microcarriers following apoptosis prevention by galactose/glutamine feeding. J Biotechnol.

[28] Li Y, Chen Y, Zhang J (2010). Protective effect of glutamine-enriched early enteral nutrition on intestinal mucosal barrier injury after liver transplantation in rats. Am J Surg.

[29] Rhoads JM, Argenzio RA, Chen W (1997). L-glutamine stimulates intestinal cell proliferation and activates mitogen-activated protein kinases. Am J Physiol.

[30] Biolo G, Zorat F, Antonione R, Ciocchi B (2005). Muscle glutamine depletion in the intensive care unit. Int J Biochem Cell Biol.

[31] Barbosa FF, Ferreira AS, Gattás G (2007). Spray dry blood plasma levels in diets for piglets weaned at 21 days of age. R Bras Zootec.

[32] Bertol TM, Santos Filho JI, Ludke JV (2000). Dietary lactose levels for weaning pigs. Rev Bras Zootec.

[33] Mahan DC (1992). Efficacy of dried whey and its lactalbumin and lactose components at two dietary lysine levels on postweaning pig performance and nitrogen balance. J Anim Sci.

[34] Pond WG, Mersmann HJ (1990). Differential compensatory growth in swine following control of feed intake by a high-alfalfa diet fed *ad libitum* or by limited feed. J Anim Sci.

[35] Lovatto PA, Sauvant D, Noblet J, Dubois S, van Milgen J (2006). Effects of feed restriction and subsequent refeeding on energy utilization in growing pigs. J Anim Sci.

[36] Watford M, Kutschenko M, Nogueira ET (2011). Optimal dietary glutamine for growth and development. Rev Bras Zootec.

